# Evaluation of the pSOFA Score in a Pediatric Intensive Care Unit and Its Discriminative Performance Compared with PRISM 4 and PIM 3

**DOI:** 10.3390/healthcare14193162

**Published:** 2026-09-24

**Authors:** Ferhat Sarı, Gokhan Ceylan, Ozlem Sarac, Gulhan Atakul, Sevgi Topal, Mustafa Colak, Ekin Soydan, Hasan Agin, Aynur Aliyeva

**Affiliations:** 1 Pediatric Intensive Care Unit, Bezmialem Vakif University, Fatih, Istanbul 34093, Turkey; 2 Research and New Technology Department, Hamilton Medical AG, 7402 Bonaduz, Switzerland; 3 Pediatric Intensive Care Unit, Dr. Behcet Uz Children’s Hospital, University of Health Sciences, Izmir 35210, Turkey; 4 Pediatric Intensive Care Unit, Acibadem Altunizade Hospital, Acibadem University, Istanbul 34662, Turkey; 5 Pediatric Intensive Care Unit, Cam Sakura Research and Training Hospital, University of Health Sciences, Istanbul 34480, Turkey; 6 Pediatric Intensive Care Unit, Aydin Maternity and Children’s Hospital, Aydın 09020, Turkey; 7 Division of Otolaryngology, Department of Surgery, Denver Health Medical Center, Denver, CO 80204, USA; 8 Neuroscience Doctoral Program, Yeditepe University, Istanbul 34755, Turkey

**Keywords:** pSOFA, PRISM 4, PIM 3, mortality prediction, prognostic scores

## Abstract

**Highlights:**

**What are the main findings?**

- pSOFA remained independently associated with PICU mortality in a cohort of 1440 critically ill children, with mortality increasing markedly across higher pSOFA strata.
- pSOFA showed good discrimination for mortality with an AUC of 0.807 (95% CI 0.773–0.841).
- PRISM 4 had significantly greater discrimination than pSOFA (ΔAUC 0.040, *p* = 0.011), whereas the difference between pSOFA and PIM 3 was not statistically significant (*p* = 0.098).
- In multivariable analysis, higher pSOFA remained independently associated with mortality, and ROC analysis assessed its discriminative performance; higher serum albumin was independently associated with lower mortality.
- PRISM 4 demonstrated the highest discrimination, whereas pSOFA offered a practical bedside assessment of organ dysfunction using routinely available clinical and laboratory parameters.

**What are the implications of the main findings?**

- pSOFA may provide a practical bedside measure of organ dysfunction and mortality risk using routinely available clinical and laboratory data.
- It should be considered complementary to, rather than a replacement for, established mortality prediction models such as PRISM 4 and PIM 3.
- Prospective multicenter external validation is needed before broader clinical implementation, because humanity apparently still insists we prove things beyond a single center.

**Abstract:**

**Objectives**: We aimed to evaluate the performance of the age-adapted Pediatric Sequential Organ Failure Assessment (pSOFA) score, as a predictor of mortality in patients admitted to pediatric intensive care units (PICUs), and to compare it with the Pediatric Risk of Mortality 4 (PRISM 4) and Pediatric Index of Mortality 3 (PIM 3) scores. **Methods**: This single-center, retrospective study analyzed data from 1440 eligible PICU admissions to a tertiary PICU. The pSOFA, PRISM 4, and PIM 3 scores were calculated retrospectively for each patient. **Results**: The overall mortality was 13.1%, and the median length of stay was 11 days. The median (IQR) pSOFA score was significantly higher in non-survivors than in survivors [6 (4–10) vs. 3 (2–4), *p* < 0.001]. Mortality increased significantly with increasing pSOFA scores (*p* < 0.001). The area under the receiver operating characteristic curve (AUC) values for pSOFA, PRISM 4, and PIM 3 were 0.807, 0.847, and 0.781, respectively. The pSOFA score exhibited a significant positive correlation with both PRISM 4 and PIM 3 (*p* < 0.001). PRISM 4 and PIM 3 demonstrated evidence of miscalibration in this cohort, with observed mortality exceeding mean predicted mortality for both models. **Conclusions**: pSOFA demonstrated good discrimination for PICU mortality and a strong association with mortality in this single-center retrospective cohort. PRISM 4 showed significantly greater discrimination than pSOFA, whereas pSOFA and PIM 3 did not differ significantly. These findings support further prospective multicenter evaluation before broader clinical implementation.

## 1. Introduction

Prognostic scoring systems were introduced in the 1980s to improve risk stratification, assess disease severity, compare intra- and inter-institutional outcomes, optimize quality assessment, and refine cost–benefit analyses in intensive care units (ICUs) [1]. Over time, these systems have been meticulously adapted to various pediatric age groups. When calculated within specified timeframes, each scoring model aims to reflect the clinical status and probability of survival with maximum accuracy.

Scoring systems in the ICU setting were initially developed for adult populations and were unsuitable for pediatric use. Consequently, specialized pediatric scoring systems were either adapted from adult models or designed entirely anew. Today, numerous scoring systems are utilized for critically ill children. Extensive research is conducted to determine which scoring model performs best, thereby facilitating the ongoing refinement and updating of existing systems [2,3].

The Pediatric Index of Mortality (PIM) score was designed as a simpler, faster, and earlier-applicable system than the PRISM 3 score [2]. Unlike PRISM, the PIM scoring system incorporates three critical variables recorded at PICU admission: post-surgical or post-procedural status, post-cardiac bypass status, and high-risk diagnoses. Over time, while PIM 2 proved successful in predicting mortality and survival, it was found inadequate for prognosticating chronic outcomes. To address this, PIM 3 was introduced in 2013 as an updated version of PIM 2, utilizing data from pediatric patients admitted to six PICUs across four countries (Australia, New Zealand, Ireland, and the United Kingdom) [4,5].

The Pediatric Risk of Mortality (PRISM) score is a physiology-based measure of disease severity widely used in clinical practice, calculated from frequently monitored physiological variables. The Eunice Kennedy Shriver National Institute of Child Health and Human Development Collaborative Pediatric Critical Care Research Network (CPCCRN) developed the PRISM 4 score in 2015. It was revised from the PRISM 3 score, drawing insights from the Trichotomous Outcome Prediction in Critical Care (TOPICC) study [6,7,8,9]. PICU outcomes are multifactorial, with age, organ dysfunction, infection, ventilation, nutritional status, body weight, and procedure-related factors all potentially contributing to clinical risk; therefore, multidimensional severity scores such as pSOFA, PRISM 4, and PIM 3 are valuable for integrating these complex clinical features into structured risk assessment [10,11,12].

The SOFA score is one of the most widely used instruments to quantify organ dysfunction in critically ill adults and has recently been closely linked to sepsis diagnosis [8,9]. The clinical success of the SOFA score in adult populations stimulated investigators’ interest in adapting the model for pediatric use (The Third International Consensus Definitions for Sepsis and Septic Shock [Sepsis-3]) [9,13]. pSOFA was developed primarily to quantify organ dysfunction severity, rather than as a dedicated mortality prediction model. Therefore, comparison with PRISM 4 and PIM 3 in the present study was intended to characterize its relative discriminative performance for mortality, rather than to test formal superiority, non-inferiority, or equivalence. It was revised from the PRISM 3 score, drawing insights from the Trichotomous Outcome Prediction in Critical Care (TOPICC) study [6,7,8,9]. PRISM 4 and PIM 3 were developed specifically as pediatric mortality prediction models, whereas pSOFA was adapted from the adult SOFA framework, using age-adjusted pediatric criteria, and subsequently validated in critically ill children [4,6,13]. Recently, researchers developed the pediatric version of the SOFA score (pSOFA) and retrospectively validated it in critically ill children [4,6,14]. However, data on pediatric populations in developing countries remain sparse, and the pSOFA score requires extensive validation through prospective cohort studies or in the general population of low- to middle-income countries [15,16].

Prior work by Matics and Sanchez-Pinto, and by Schlapbach et al., validated pSOFA in single high-income-country cohorts; whether these findings generalize to a non-sepsis-restricted PICU population in an upper-middle-income setting remains unclear [5,13]. Previous studies have evaluated pSOFA predominantly in selected pediatric populations, including children with suspected infection, whereas evidence from heterogeneous general PICU populations remains more limited.

We hypothesized that higher pSOFA scores would be associated with a graded increase in mortality in a general PICU cohort. Accordingly, this study aimed to evaluate the performance of pSOFA and compare its mortality discrimination with PRISM 4 and PIM 3.

## 2. Materials and Methods

### 2.1. Study Design and Population

This retrospective study was conducted in a tertiary PICU at a training and research hospital in Turkey and included eligible patients admitted between April 2021 and May 2026. Patients aged between 1 month and 18 years who were admitted to the PICU were included. Patients aged under 1 month or over 18 years, those discharged or deceased within the first 24 h of admission, and patients with insufficient data to calculate the scoring systems were excluded from the study. Patients younger than 1 month were excluded to maintain a non-neonatal PICU cohort, given the distinct physiological characteristics and clinical care pathways of neonates compared with older infants and children.

Underlying diseases and admission diagnoses were categorized under generalized organ system disorders (e.g., respiratory, cardiac, neurological, metabolic, renal). Clinical variables that significantly impact PICU mortality—such as length of stay, duration of mechanical ventilation, microbiological culture positivity, vasoactive medication requirements, and hypoalbuminemia—were evaluated concurrently with the scoring systems.

PRISM 4, PIM 3, and pSOFA scores were utilized to assess disease severity and prognosis. PRISM 4 and PIM 3 scores were calculated using data obtained at admission. The pSOFA score was calculated for all patients using the worst parameters recorded within the first 24 h following PICU admission.

### 2.2. Components of the Scoring Systems

For the PRISM 4 calculation, laboratory variables were obtained between 2 h before and 4 h after PICU admission, whereas physiological variables were limited to data measured during the first 4 h of admission. The PRISM 4 components include age, source of admission, cardiopulmonary resuscitation within 24 h before admission, presence of cancer (acute or chronic), primary disorder of low-risk systems, and neurological/non-neurological variable sub-scores.

The PIM 3 components comprise systolic blood pressure, pupillary reaction, PaO_2/FiO_2 ratio, base excess from arterial or capillary blood gas analysis, mechanical ventilation status at any point during the first hour of admission, elective admission status, and the presence of low-, high-, or very high-risk diagnoses.

Low-risk diagnoses: Asthma, bronchiolitis, croup, obstructive sleep apnea syndrome, diabetic ketoacidosis, epilepsy.
High-risk diagnoses: Spontaneous cerebral hemorrhage, cardiomyopathy or myocarditis, hypoplastic left heart syndrome, neurodegenerative disorders, necrotizing enterocolitis.
Very high-risk diagnoses: Cardiac arrest before ICU admission, severe combined immunodeficiency, post-induction leukemia or lymphoma, bone marrow transplant recipient, liver failure.

The pSOFA score evaluates six organ systems: respiratory (PaO_2/FiO_2 or SpO_2/FiO_2), hepatic (bilirubin level), hematological (platelet count), cardiovascular (mean arterial pressure adjusted for age or vasoactive drug infusion), neurological (Glasgow Coma Scale), and renal (creatinine adjusted for age). Each system was assigned a score ranging from 0 to 4 based on the worst value recorded in the first 24 h, yielding a total score range of 0 to 24. If a variable was not measured within the 24 h window, it was considered normal (0 points), consistent with the original criteria. Unmeasured pSOFA components were assigned 0 points according to the prespecified scoring approach. Because component-level missingness could not be quantified retrospectively from the available analytic dataset, a complete-case sensitivity analysis for individual pSOFA domains was not feasible. The primary outcome was PICU mortality, defined as death occurring during the PICU stay. Secondary outcomes included PICU length of stay, duration of mechanical ventilation, microbiological culture positivity, requirement for vasoactive medications, and serum albumin level. Although pSOFA can be recalculated serially in clinical practice, the present study evaluated pSOFA using the worst values recorded during the first 24 h after PICU admission and did not assess serial pSOFA trajectories.

### 2.3. Statistical Analysis

The conformity of variables to normal distribution was evaluated using visual methods (histograms and probability plots) and analytical methods (Kolmogorov–Smirnov/Shapiro–Wilk tests). Descriptive statistics are expressed as medians (interquartile range, 25th–75th percentiles) for continuous data and as frequencies and percentages for categorical data. To evaluate factors influencing survival, the chi-square test or Fisher’s exact test was used for categorical variables, and the Mann–Whitney U test was applied to continuous variables.

The relationships between the pSOFA score and clinical variables were evaluated using Spearman’s rank correlation test to calculate correlation coefficients. The multivariable model included age, sex, serum albumin, culture positivity, and pSOFA to evaluate whether pSOFA remained independently associated with mortality after adjustment for demographic and clinically relevant covariates available in the dataset. The model was intended to assess independent association, rather than incremental predictive validity or to develop a new mortality prediction model. Because some patients had more than one eligible PICU admission, admissions were treated as the unit of analysis. A sensitivity analysis restricted to the first eligible admission per patient was performed. The diagnostic capabilities of the scoring systems were compared using receiver operating characteristic (ROC) curves. Statistical analyses were performed using IBM SPSS version 25, and a *p*-value of <0.05 was considered statistically significant.

Model discrimination was quantified using the area under the receiver operating characteristic curve (AUC), with 95% confidence intervals and pairwise comparisons of scores using the DeLong method. Model calibration was assessed using the Hosmer–Lemeshow goodness-of-fit test, the calibration slope and intercept, and the Brier score. A single omnibus chi-square test was used to compare mortality across all levels of a categorical variable simultaneously (e.g., admission diagnosis, admission source), rather than performing separate pairwise tests per category. The decision-analytic performance of each score was further examined using decision curve analysis, and discriminative performance was re-assessed within pre-specified clinical subgroups (age band, sepsis status, mechanical ventilation status, culture positivity).

## 3. Results

A total of 1440 eligible PICU admissions were included in the study. Overall PICU mortality was 13.1% (n = 189). Admission source and admission diagnosis were each compared between survivors and non-survivors using a single omnibus chi-square test across all categories (χ^2^ = 27.80, df = 5, *p* = 3.99 × 10^−5^ for admission source; χ^2^ = 54.22, df = 9, *p* = 1.71 × 10^−8^ for admission diagnosis), rather than a separate test per category. The most common admission diagnosis was respiratory disease (39.7%). Positive microbiological cultures were detected in 481 admissions (33.4%). Baseline demographic, clinical, and laboratory characteristics are summarized in Table 1.

### 3.1. Relationship Between pSOFA Score and Mortality Risk

The median pSOFA score was significantly higher among non-survivors than survivors (Table 1). When patients were divided into subgroups according to pSOFA score percentiles, the median pSOFA score was 3, and mortality increased with higher pSOFA score (*p* < 0.001). Mortality rose from 3.4% (95% CI, 2.3–4.9%) in the lowest pSOFA band (0–3) to 63.6% (95% CI, 35.4–84.8%) in patients with a pSOFA score > 15, and a Cochran–Armitage trend test confirmed a significant monotonic dose–response relationship across bands (z = 15.76, *p* < 0.0001), confirming a significant monotonic dose–response relationship between increasing pSOFA scores and mortality (Figure 1, Figure 2 and Figure 3).

In univariate logistic regression, pSOFA score, positive culture, and female sex were positively associated with mortality, while age and albumin were negatively associated. In the multivariable model, pSOFA (adjusted OR 1.352; 95% CI, 1.284–1.424; *p* < 0.001) and albumin (adjusted OR 0.471; 95% CI, 0.365–0.607; *p* < 0.001) remained independently associated with mortality after adjustment for age, sex, and culture positivity (Table 2). The model showed adequate fit (Hosmer–Lemeshow χ^2^ = 11.49, df = 8, *p* = 0.176) and no relevant multicollinearity among predictors (all variance inflation factors < 1.2) (Table 2).

The AUC for predicting mortality was 0.807 (95% CI, 0.773–0.841) for pSOFA, 0.847 (95% CI, 0.813–0.881) for PRISM 4, and 0.781 (95% CI, 0.747–0.816) for PIM 3 (Table 3, Figure 1 and Figure 2). Pairwise DeLong testing showed that PRISM 4 discriminated significantly better than pSOFA (ΔAUC = 0.040; 95% CI, 0.009–0.071; *p* = 0.011), whereas the difference between pSOFA and PIM 3 did not reach statistical significance (ΔAUC = 0.025; 95% CI, −0.005 to 0.055; *p* = 0.098); PRISM 4 also outperformed PIM 3 (ΔAUC = 0.065; 95% CI, 0.032–0.099; *p* < 0.001) (Table 3, Figure 1).

### 3.2. Calibration of the Scoring Systems

Both PRISM 4 and PIM 3 demonstrated evidence of miscalibration in this cohort (Hosmer–Lemeshow *p* < 0.001 for both). Observed mortality exceeded mean predicted mortality for both models, indicating overall underprediction of absolute mortality risk, while calibration slopes below 1 (0.87 for PRISM 4 and 0.59 for PIM 3) indicated overly extreme predictions, particularly for PIM 3. The corresponding calibration intercepts and Brier scores are provided in Supplementary Tables S1 and S2.

### 3.3. Subgroup Discrimination and Decision Curve Analysis

Lower AUCs were observed for all three scores among mechanically ventilated and culture-positive patients compared with the overall cohort; however, no formal statistical comparisons or interaction testing between subgroups were performed, and these differences should therefore be interpreted descriptively (Figure 4 and Figure 5). Decision curve analysis showed positive net benefit for all three scores relative to the treat-none strategy across the evaluated threshold range. Compared with the treat-all strategy, net benefit increased from about 3% for pSOFA, 6% for PRISM 4, and 10% for PIM 3, and remained greater at higher threshold probabilities. These findings indicate potential decision-analytic utility under the assumptions of the analysis and should not be interpreted as evidence of improved clinical outcomes or as support for treatment decisions based on these scores alone (Figure 4 and Figure 5).

### 3.4. Correlation of pSOFA Score with Clinical Parameters

Mortality by pSOFA risk band and score discrimination within pre-specified clinical subgroups are shown in Table 4. Correlation analysis demonstrated that the pSOFA score was significantly and positively correlated with PRISM 4 (r_s = 0.625), PIM 3 (r_s = 0.580), duration of mechanical ventilation (r_s = 0.332), and PICU length of stay (LOS) (r_s = 0.177) (*p* < 0.001, Table 4). Conversely, age and serum albumin levels exhibited significant negative correlations with the pSOFA score.

Full statistical details for the admission-category omnibus tests, calibration analysis, decision curve data, and sensitivity analyses are provided in Supplementary Tables S1–S4.

## 4. Discussion

In this study, the performance of the pSOFA score in predicting mortality was evaluated and compared with the PRISM 4 and PIM 3 scores using data from 1440 critically ill patients monitored in a tertiary PICU. The primary findings of our study demonstrate that the pSOFA score is strongly associated with mortality, with mortality increasing in a graded manner as the score rises. Furthermore, pSOFA remained independently associated with mortality in multivariable analysis, while its discriminative performance was assessed separately by ROC analysis. PRISM 4 demonstrated significantly greater discrimination than pSOFA, whereas the difference between pSOFA and PIM 3 was not statistically significant. These findings underscore the prognostic significance of organ dysfunction in pediatric intensive care, while supporting careful consideration of the potential decision-analytic value of pSOFA.

One of the key predictors of mortality in intensive care practice is the presence and severity of multiple organ dysfunction syndrome (MODS) [17,18].

The adult SOFA score has been widely implemented for decades to standardize the assessment of organ dysfunction. Notably, the Sepsis-3 consensus report emphasized that an increase in the SOFA score is closely linked to mortality, placing organ dysfunction at the core of sepsis definition [19]. However, due to age-dependent physiological variations in children, adult scoring systems cannot be applied directly to pediatric populations, thereby justifying the need for an age-adapted pSOFA score [20,21].

In our study, the pSOFA score had an AUC of 0.807 for mortality prediction. This result is highly consistent with the original validation study by Matics and Sanchez-Pinto, which reported excellent discriminative power for hospital mortality and performance, comparable to the PELOD score [13]. Similarly, Schlapbach et al. reported high predictive accuracy for organ dysfunction-based scores in pediatric sepsis [5]. Our findings extend these observations by showing that pSOFA demonstrated good mortality discrimination across a heterogeneous general PICU population.

A notable finding was the graded increase in mortality across predefined pSOFA risk bands. Mortality increased from 3.4% in patients with pSOFA scores of 0–3 to 63.6% in those with scores >15, consistent with the significant monotonic trend observed across ordered risk categories. This clearly highlights the profound impact of cumulative organ dysfunction on survival. In the adult literature, Ferreira et al. demonstrated that serial SOFA measurements strongly correlate with mortality. Our findings are consistent with a graded association between pSOFA and mortality in pediatric critical illness [22].

Multivariate logistic regression showed that the pSOFA score remains an independent risk factor for mortality, confirming that organ dysfunction has prognostic value independent of age, sex, and culture positivity. This finding is particularly important because mortality in critical illness frequently reflects the aggregate burden of cascading organ failures, rather than a single primary diagnosis. These findings support the prognostic relevance of organ dysfunction across heterogeneous disease etiologies [23].

The finding that PRISM 4 achieved the highest discriminative performance in the ROC analysis (AUC = 0.847) was anticipated. PRISM 4 is a comprehensive model that integrates a wide range of physiological variables, laboratory parameters, and pre-existing clinical risk factors. Pollack et al. [6] similarly reported excellent mortality prediction for PRISM 4 in their development cohort [24]. However, PRISM 4’s data-intensive requirements and complex algorithmic calculations can limit its bedside practicality. In contrast, pSOFA relies on routine parameters easily obtained in daily clinical practice. Although pSOFA demonstrated good discrimination, PRISM 4 showed significantly greater discriminative performance in this cohort (AUC 0.847 vs. 0.807; DeLong *p* = 0.011). The potential advantage of pSOFA, therefore, lies in its practical simplicity and routine bedside availability, rather than in its equivalent mortality discrimination.

Formal pairwise comparison using the DeLong test indicated that PRISM 4’s discriminative advantage over pSOFA was statistically significant (*p* = 0.011), while pSOFA’s numerically higher AUC relative to PIM 3 did not reach statistical significance in this cohort (*p* = 0.098). The difference in discrimination between pSOFA and PIM 3 was not statistically significant in this cohort (*p* = 0.098); however, this finding should not be interpreted as evidence of formal equivalence.

The lower discriminative performance of PIM 3 in our cohort (AUC = 0.781) is consistent with several prior reports [7]. While PIM 3 is primarily constrained to physiological data collected strictly at PICU admission, pSOFA captures the worst manifestations of evolving organ dysfunction over the first 24 h. Because pSOFA incorporates information over a longer observation window than the admission-based scores, the comparison does not reflect equivalent information horizons, which may contribute to the observed discrimination. This advantage may be amplified in centers with prolonged referral chains, where patients are admitted at advanced stages of illness. This interpretation should be treated as hypothesis-generating, rather than causal, since a patient’s trajectory over the 24 h pSOFA window reflects the effect of PICU treatment, as well as admission severity, and our retrospective design cannot separate the two. The origin of our data from Turkey, an upper-middle-income developing country, adds valuable regional diversity to the existing literature, which is predominantly heavily weighted toward high-income nations [25,26,27,28].

Furthermore, pSOFA showed positive correlations with duration of mechanical ventilation (r = 0.332) and PICU length of stay (r = 0.177). These associations were modest, particularly for length of stay, and should therefore be interpreted as descriptive relationships with measures of resource utilization, rather than evidence that pSOFA can guide resource allocation or institutional planning [29].

Another striking finding was the independent negative association of serum albumin levels with mortality (Adjusted OR: 0.471). Hypoalbuminemia in critical illness serves as a biochemical surrogate for systemic inflammation, capillary leak syndrome, malnutrition, and hepatic synthetic impairment. Numerous pediatric intensive care studies have substantiated that reduced albumin levels predict increased mortality and prolonged stay [30]. In the present analysis, serum albumin was considered an exploratory covariate and remained independently associated with mortality. However, this study was not designed to assess whether albumin provides incremental predictive value or should be incorporated into a new prognostic model.

The positive correlation between the pSOFA score and both PRISM 4 (r = 0.625) and PIM 3 (r = 0.580) confirms that these scoring models share a common baseline for measuring disease severity. However, the moderate magnitude of these correlation coefficients suggests that each model targets distinct clinical dimensions: PRISM 4 integrates physiological derangement with premorbid risk factors, whereas pSOFA directly quantifies dynamic, active organ dysfunction. Consequently, several investigators have proposed that combining these models might further enhance prognostic precision [14].

### Study Limitations

The retrospective design of this study inherently limits the ability to determine direct causal relationships. The single-center framework may restrict the generalizability of our findings to alternative healthcare settings. Additionally, the standard protocol of assigning a normal score (0 points) to missing pSOFA parameters may have introduced a conservative bias, potentially underestimating the true severity of organ dysfunction in some patients. Conversely, the major strengths of this study include its substantial sample size of 1440 patients, a highly heterogeneous patient population, and the implementation of robust, real-world clinical data.

Another limitation is the inability to quantify component-level missingness. Assigning 0 points to unmeasured pSOFA components may have underestimated organ dysfunction in some patients if the absence of measurement did not correspond to normal physiology.

Exclusion of patients who died or were discharged within the first 24 h may have introduced selection bias by excluding patients at the extremes of early clinical severity or recovery. Changes in PICU management practices during the study period may also have influenced outcomes. In addition, this was a single-center evaluation without external validation. Importantly, the scoring systems were not based on equivalent information windows: pSOFA incorporated the worst physiological and laboratory values during the first 24 h, whereas PRISM 4 and PIM 3 relied predominantly on data obtained at or shortly after admission. This difference may influence comparative discrimination because pSOFA incorporates information arising during the early clinical course and treatment.

Furthermore, both PRISM 4 and PIM 3 showed significant miscalibration in our cohort (Hosmer–Lemeshow *p* < 0.001 for each). Observed mortality exceeded mean predicted mortality for both models, indicating overall underprediction of absolute mortality risk, while calibration slopes below 1 suggested overly extreme predictions across the risk range, particularly for PIM 3. This likely reflects case mix differences between our single-center Turkish cohort and the multinational, high-income-country populations in which these scores were originally developed and calibrated, and underscores that AUC alone does not capture a model’s absolute risk accuracy.

Future research should explore integrating artificial intelligence and machine-learning approaches with established severity scores, such as pSOFA, PRISM 4, and PIM 3, to improve individualized risk prediction and support more data-driven PICU management [31,32,33].

## 5. Conclusions

In this single-center retrospective cohort, pSOFA demonstrated good discrimination and a strong graded association with PICU mortality. PRISM 4 showed significantly greater discrimination than pSOFA, whereas the difference between pSOFA and PIM 3 was not statistically significant. These findings support pSOFA as a complementary measure of organ dysfunction, rather than a replacement for established mortality prediction models. Prospective multicenter external validation is required before broader clinical implementation.

## Figures and Tables

**Figure 1:**
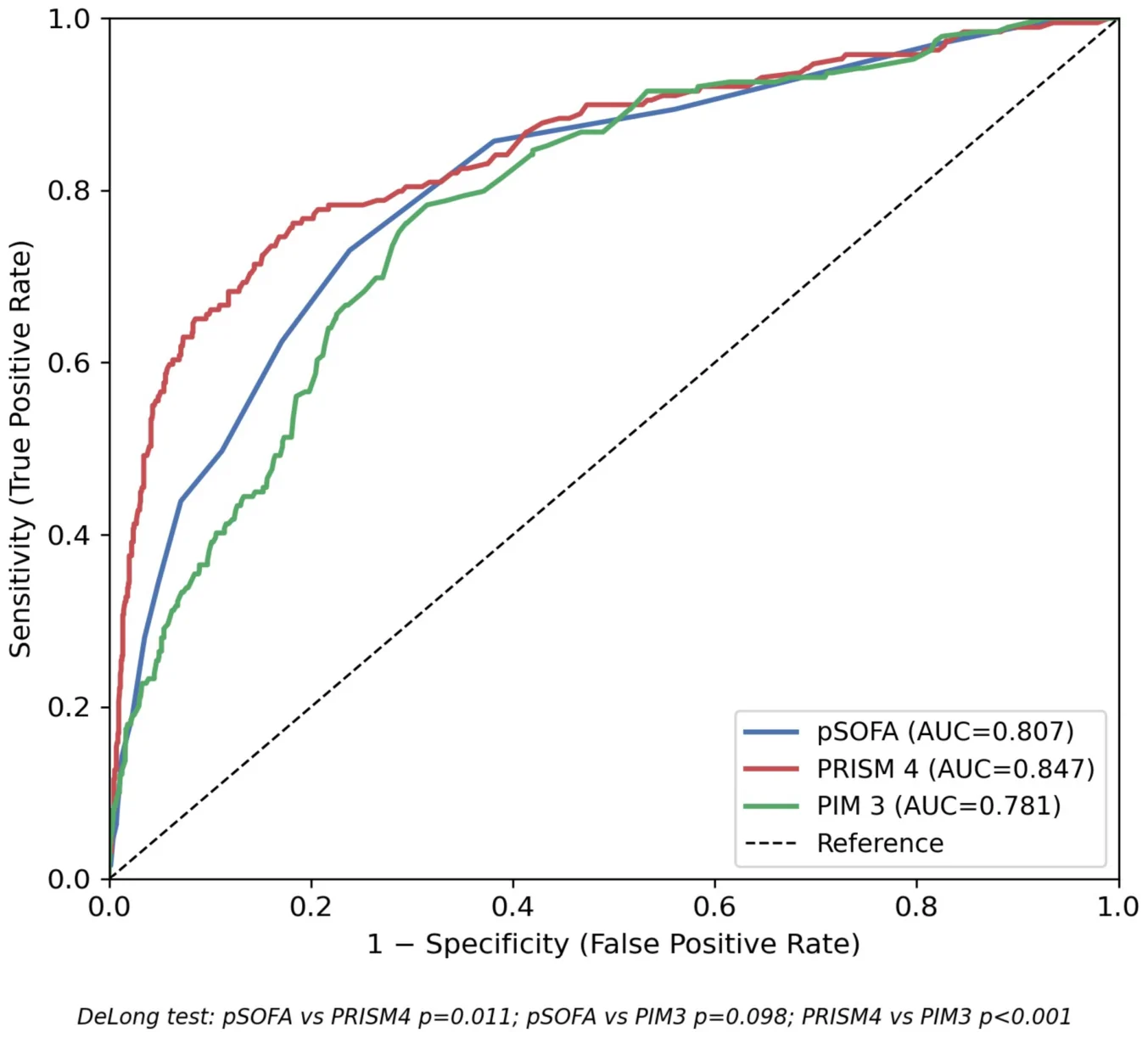
ROC Curves for pSOFA, PRISM 4, and PIM 3 (n = 1440).

**Figure 2:**
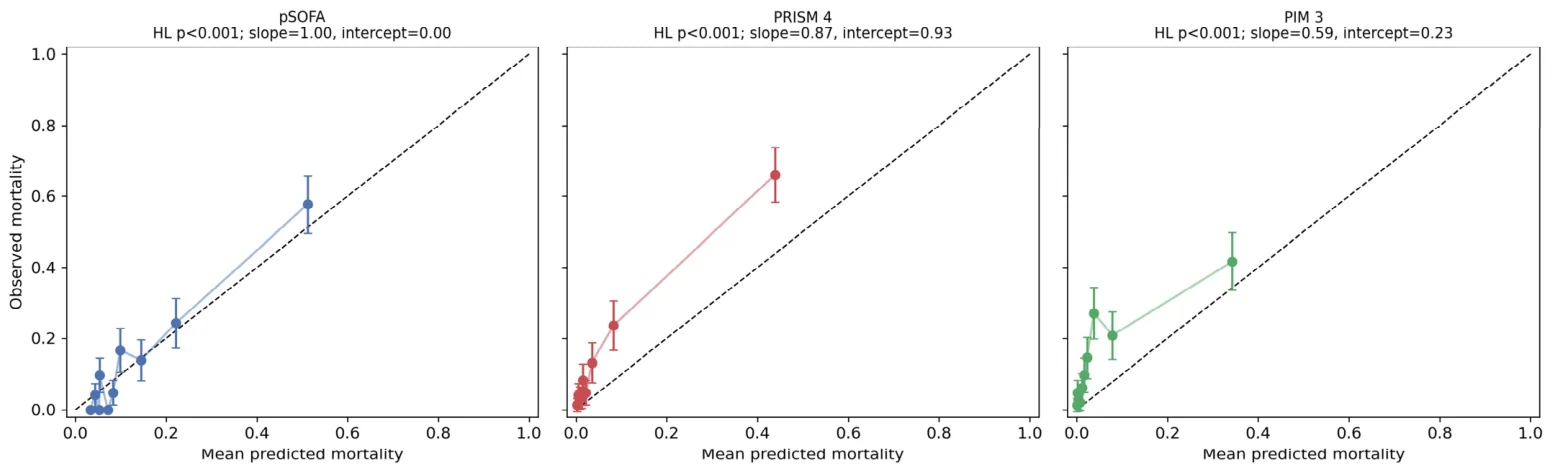
Calibration plots by decile of predicted risk (n = 1440).

**Figure 3:**
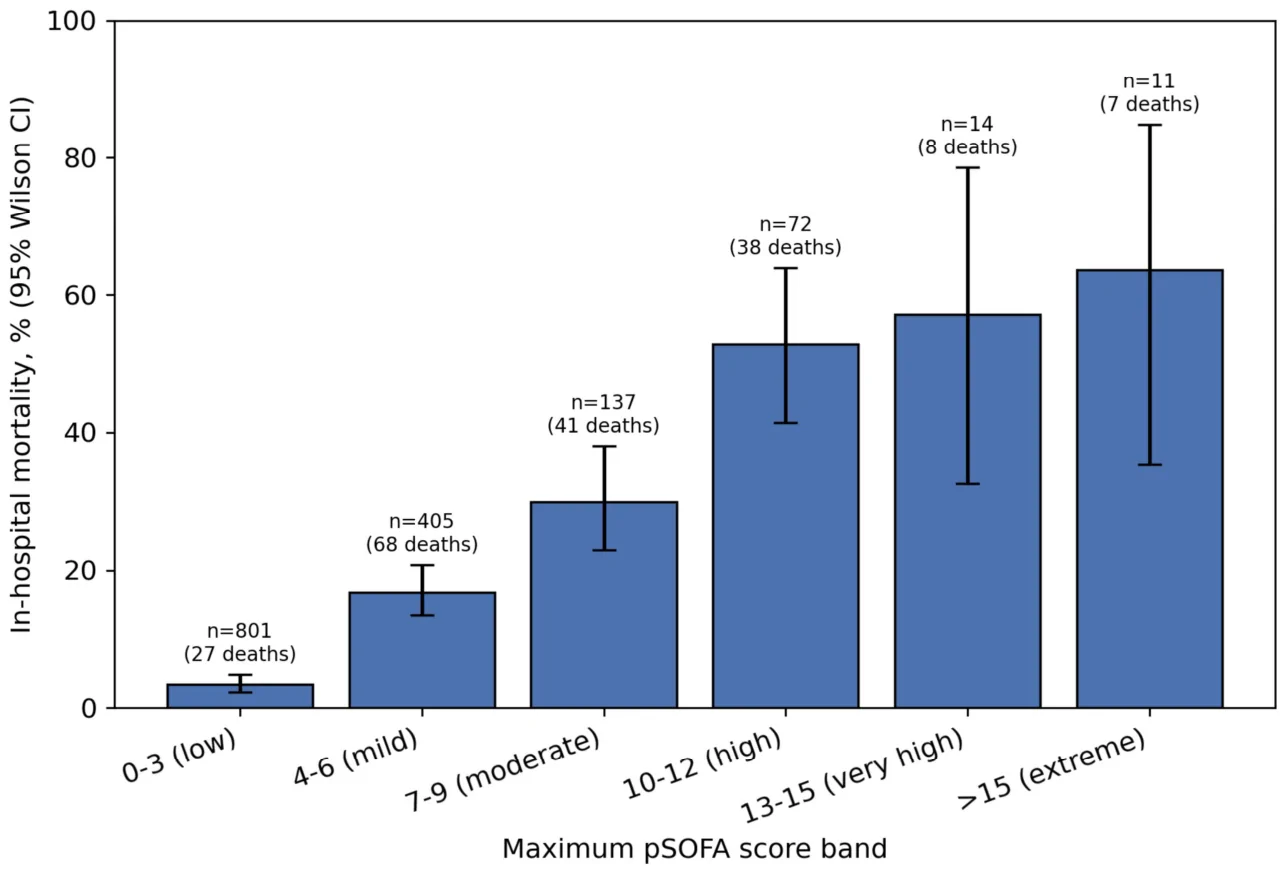
Mortality by pSOFA risk band.

**Figure 4:**
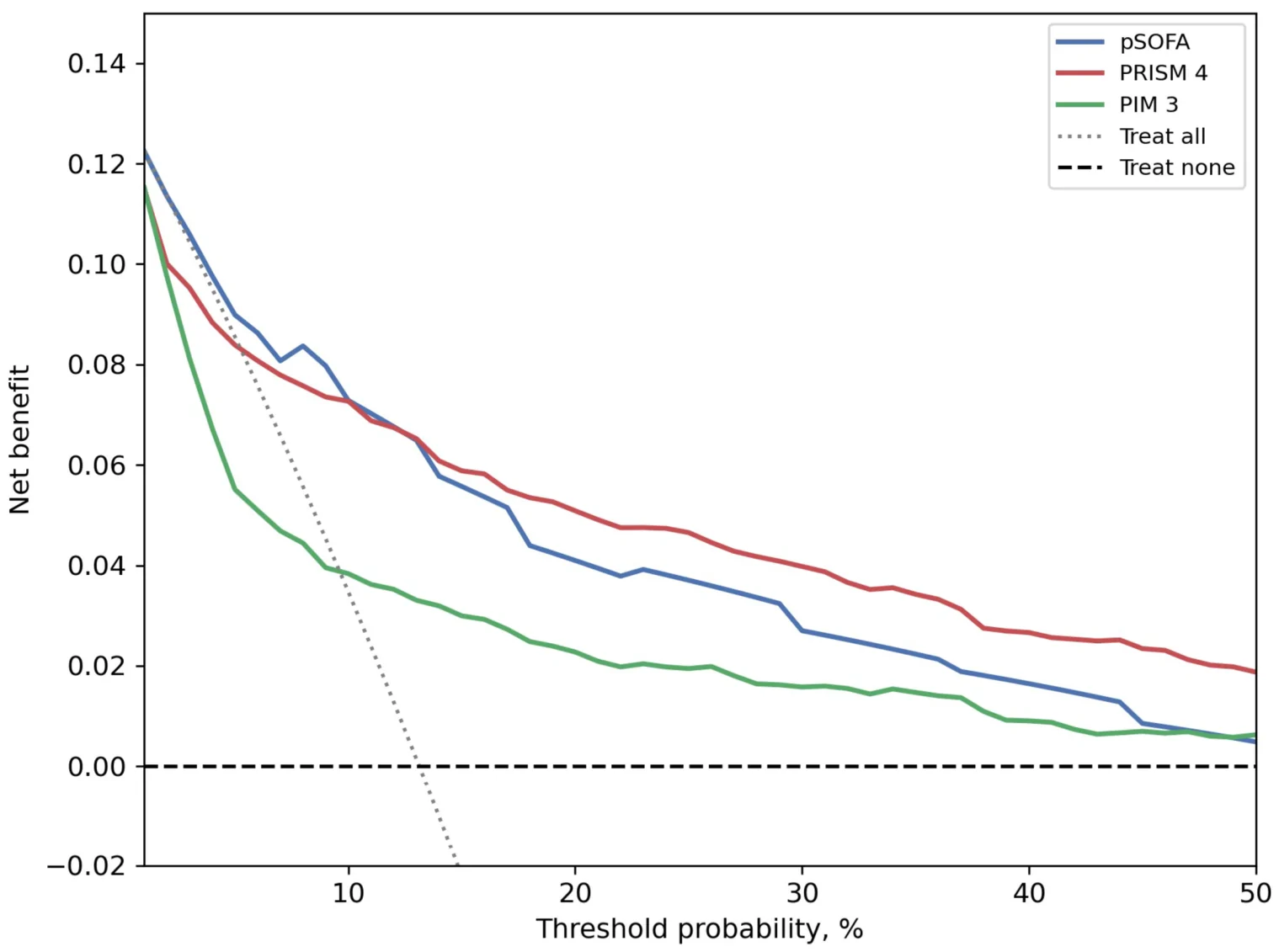
Decision curve analysis.

**Figure 5:**
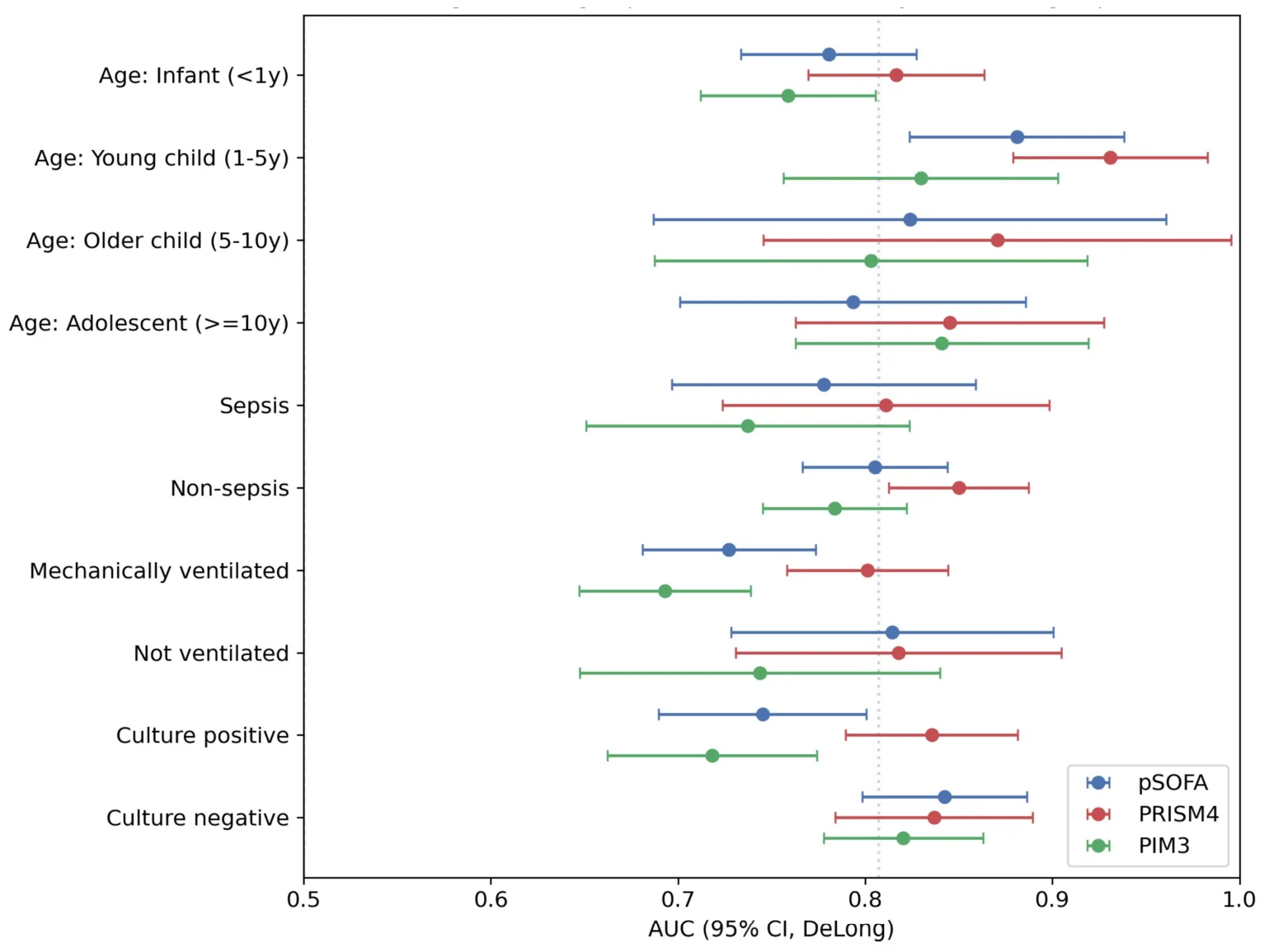
Subgroup discrimination (AUC) by clinical subgroup.

**Table 1:** Demographic, clinical, and laboratory characteristics.

Characteristic	Total (n = 1440)	Survivors (n = 1251)	Non-survivors (n = 189)	*p*
Age, months, median (IQR)/mean ± SD	13.0 (8.0–72.0)/46.16 ± 56.28	18.0 (8.0–72.0)/47.24 ± 56.26	11.0 (5.0–48.0)/39.03 ± 56.05	<0.001
Male sex, n (%)	721 (51.4%)	639 (52.5%)	82 (44.3%)	0.047
***Admission source, n (%)—overall p***				**<0.001**
Emergency dept.	580 (40.4%)	519 (41.6%)	61 (32.3%)	
Home	35 (2.4%)	33 (2.6%)	2 (1.1%)	
Inpatient ward	335 (23.3%)	265 (21.2%)	70 (37.0%)	
Operating room	7 (0.5%)	7 (0.6%)	0 (0.0%)	
Other hospital transfer	470 (32.7%)	417 (33.4%)	53 (28.0%)	
Surgical unit	10 (0.7%)	7 (0.6%)	3 (1.6%)	
***Admission diagnosis, n (%)—overall p***				**<0.001**
Respiratory	573 (39.8%)	497 (39.7%)	76 (40.2%)	
Neurologic	242 (16.8%)	223 (17.8%)	19 (10.1%)	
Sepsis	172 (11.9%)	136 (10.9%)	36 (19.0%)	
Cardiac	100 (6.9%)	81 (6.5%)	19 (10.1%)	
Shock	95 (6.6%)	75 (6.0%)	20 (10.6%)	
Intoxication	92 (6.4%)	92 (7.4%)	0 (0.0%)	
Metabolic/Endocrine	35 (2.4%)	29 (2.3%)	6 (3.2%)	
Hematologic/Oncologic	23 (1.6%)	15 (1.2%)	8 (4.2%)	
Postsurgical recovery	8 (0.6%)	8 (0.6%)	0 (0.0%)	
Other	100 (6.9%)	95 (7.6%)	5 (2.6%)	
Mechanical ventilation, n (%)	577 (40.1%)	421 (33.7%)	156 (82.5%)	<0.001
MV duration, days, median (IQR)	0.0 (0.0–5.0)	0.0 (0.0–3.0)	4.0 (1.0–18.0)	<0.001
Vasoactive infusion, n (%)	458 (31.9%)	302 (24.2%)	156 (83.9%)	<0.001
Positive microbiological culture, n (%)	481 (33.4%)	389 (31.1%)	92 (48.9%)	<0.001
Albumin, g/dL, median (IQR)/mean ± SD	3.7 (3.2–4.1)/3.61 ± 0.68	3.7 (3.3–4.1)/3.68 ± 0.65	3.2 (2.6–3.7)/3.16 ± 0.74	<0.001
pSOFA score, median (IQR)/mean ± SD	3.0 (2.0–5.0)/3.94 ± 3.08	3.0 (2.0–4.0)/3.43 ± 2.56	6.0 (4.0–10.0)/7.29 ± 4.01	<0.001
PRISM 4 score, median (IQR)/mean ± SD	3.0 (2.0–8.0)/5.35 ± 5.67	3.0 (1.0–6.0)/4.45 ± 4.69	10.0 (6.0–16.0)/11.34 ± 7.62	<0.001
PRISM 4 predicted mortality, %, median (IQR)	1.4 (0.8–3.4)	1.2 (0.7–2.4)	15.0 (3.4–44.0)	<0.001
PIM 3 predicted mortality, %, median (IQR)	1.4 (0.3–3.8)	1.2 (0.2–2.8)	4.6 (2.3–21.0)	<0.001

Continuous variables: median (IQR) [age, albumin, pSOFA, PRISM4 also mean ± SD]. Categorical: n (%). Tests: Mann–Whitney U (continuous); chi-square (categorical). Admission source and admission diagnosis each use a single OVERALL chi-square test across all categories (χ^2^ = 27.80, df = 5, *p* = 3.99 × 10^−5^ for source; χ^2^ = 54.22, df = 9, *p* = 1.71 × 10^−8^ for diagnosis), rather than a separate *p* per row.

**Table 2:** Logistic regression for prediction of mortality.

Variable	Univariate OR (95% CI)	*p*	Adjusted OR (95% CI)	*p*	VIF
Age (months)	0.997 (0.994–1.000)	0.066	0.998 (0.994–1.001)	0.185	1.02
Female sex	1.377 (1.012–1.873)	0.042	1.133 (0.798–1.608)	0.486	1.01
Albumin (g/dL)	0.338 (0.268–0.426)	<0.001	0.471 (0.365–0.607)	<0.001	1.12
Positive culture	2.124 (1.558–2.895)	<0.001	1.402 (0.981–2.002)	0.063	1.09
pSOFA score	1.399 (1.330–1.472)	<0.001	1.352 (1.284–1.424)	<0.001	1.08

Multivariable model: died ~ age + sex + albumin + positive culture + pSOFA. Hosmer–Lemeshow goodness-of-fit: χ^2^ = 11.485, df = 8, *p* = 0.176. McFadden pseudo-R^2^ = 0.228; AIC = 873.6. VIF, variance inflation factor (all < 1.2, no multicollinearity). Independently re-derived in base R (glm).

**Table 3:** ROC performance and pairwise DeLong comparison.

Score	AUC (95% CI)	Youden Cutoff	Sens.	Spec.	PPV	NPV	LR+/LR−	HL Calibration *p*
pSOFA	0.807 (0.773–0.841)	5.00	73.0%	76.2%	31.7%	94.9%	3.07/0.35	<0.001
PRISM 4	0.847 (0.813–0.881)	3.30	76.2%	81.8%	38.7%	95.8%	4.18/0.29	<0.001
PIM 3	0.781 (0.747–0.816)	2.10	78.3%	68.5%	27.3%	95.4%	2.49/0.32	<0.001
**Comparison**		**ΔAUC (95% CI)**			**Z**		**DeLong *p***	
pSOFA vs. PRISM 4		−0.0400 (−0.0709 to −0.0091)			−2.538		0.011	
pSOFA vs. PIM 3		0.0254 (−0.0047 to 0.0554)			1.654		0.098	
PRISM 4 vs. PIM 3		0.0653 (0.0315 to 0.0992)			3.781		<0.001	

AUC and 95% CI via DeLong method. Youden cutoff, sensitivity/specificity/PPV/NPV/LR from the raw pSOFA score and from the PRISM 4/PIM 3 percent-mortality outputs. HL calibration *p*: Hosmer–Lemeshow goodness-of-fit for each score’s own predicted risk output (*p* < 0.05).

**Table 4:** (**a**). Mortality by pSOFA risk band. (**b**). Subgroup discrimination (AUC) by clinical subgroup.

(a)					
pSOFA Band	N	Deaths	Mortality %	95% CI Lower	95% CI Upper
0–3 (low)	801	27	3.4%	2.3%	4.9%
4–6 (mild)	405	68	16.8%	13.5%	20.7%
7–9 (moderate)	137	41	29.9%	22.9%	38.1%
10–12 (high)	72	38	52.8%	41.4%	63.9%
13–15 (very high)	14	8	57.1%	32.6%	78.6%
>15 (extreme)	11	7	63.6%	35.4%	84.8%
(**b**)					
**Subgroup**	**N**	**Deaths**	**pSOFA AUC (95% CI)**	**PRISM 4 AUC (95% CI)**	**PIM 3 AUC (95% CI)**
Age: Infant (<1 y)	698	117	0.781 (0.734–0.827)	0.817 (0.769–0.864)	0.759 (0.712–0.806)
Age: Young child (1–5 y)	366	30	0.881 (0.824–0.938)	0.931 (0.879–0.983)	0.830 (0.756–0.903)
Age: Older child (5–10 y)	177	17	0.824 (0.687–0.961)	0.871 (0.746–0.996)	0.803 (0.688–0.919)
Age: Adolescent (>=10 y)	199	25	0.793 (0.701–0.886)	0.845 (0.763–0.928)	0.841 (0.763–0.919)
Sepsis	172	36	0.778 (0.697–0.859)	0.811 (0.724–0.898)	0.737 (0.651–0.824)
Non-sepsis	1268	153	0.805 (0.766–0.844)	0.850 (0.813–0.887)	0.784 (0.745–0.822)
Mechanically ventilated	577	156	0.727 (0.681–0.773)	0.801 (0.758–0.844)	0.693 (0.647–0.739)
Not ventilated	863	33	0.815 (0.728–0.901)	0.818 (0.731–0.905)	0.744 (0.647–0.840)
Culture-positive	482	93	0.745 (0.690–0.801)	0.836 (0.790–0.882)	0.718 (0.662–0.774)
Culture-negative	958	96	0.842 (0.799–0.886)	0.837 (0.784–0.890)	0.820 (0.778–0.863)

Wilson 95% CI. Cochran–Armitage trend test for monotonic dose–response across ordered bands: z = 15.76, *p* < 0.0001. AUC (DeLong 95% CI) computed independently within each subgroup. Discrimination for all three scores is numerically lower among mechanically ventilated and culture-positive patients.

## Data Availability

The data supporting the findings of this study are available from the corresponding author upon reasonable request. The data are not publicly available due to privacy and ethical restrictions.

## Supplementary Materials

The following supporting information can be downloaded at: https://www.mdpi.com/article/10.3390/healthcare14193162/s1, Table S1. Admission-category omnibus chi-square tests. Table S2. Calibration analysis data. Table S3. Decision curve analysis. Table S4. Sensitivity analyses.

## Abbreviations

AUC	area under the receiver operating characteristic curve
CI	confidence interval
DCA	decision curve analysis
ICU	intensive care unit
LOS	length of stay
MODS	multiple organ dysfunction syndrome
MV	mechanical ventilation
OR	odds ratio
PICU	pediatric intensive care unit
PIM 3	Pediatric Index of Mortality 3
PRISM 4	Pediatric Risk of Mortality 4
pSOFA	Pediatric Sequential Organ Failure Assessment
ROC	receiver operating characteristic
SOFA	Sequential Organ Failure Assessment
SpO_2_/FiO_2_	peripheral oxygen saturation to fraction of inspired oxygen ratio
PaO_2_/FiO_2_	arterial oxygen partial pressure to fraction of inspired oxygen ratio
VIF	variance inflation factor
