# Supplementary material for: Evaluation of the pSOFA Score in a Pediatric Intensive Care Unit and Its Discriminative Performance Compared with PRISM 4 and PIM 3
Source: Healthcare (Basel). 2026 Sep 24;14(19):3162. doi: 10.3390/healthcare14193162 (PMC13664861; doi:10.3390/healthcare14193162)
Supplement: Supplementary file 1 [file healthcare-14-03162-s001.zip › healthcare-4534461-supplementary.pdf]

Supplementary Materials

Evaluation of the pSOFA Score in a Pediatric Intensive Care Unit and Its Discriminative Performance Compared with PRISM 4 and PIM 3

Running Title: Evaluation of the pSOFA Score

Ferhat Sarı, Gokhan Ceylan, Ozlem Sarac, Gulhan Atakul, Sevgi Topal, Mustafa Colak, Ekin Soydan, Hasan Agin and Aynur Aliyeva

Supplementary tables

Table S1. Admission-category omnibus chi-square tests
Table S2. Calibration analysis data
Table S3. Decision curve analysis
Table S4. Sensitivity analyses

Table S1. Admission-category omnibus chi-square tests

Test	Chi-square	df	P value	Min expected cell count
Admission diagnosis (10 categories) vs mortality	54.22	9	1.71e-08	1.05*
Admission source (6 categories) vs mortality	27.798	5	3.99e-05	0.921

Note: A single Pearson chi-square test was run across ALL categories simultaneously (the standard "omnibus" test)
*Pearson chi-square valid per Cochran rule (min expected count = 1.05)

Contingency table (admission diagnosis x mortality):

Admission diagnosis	Survived	Died
Cardiac	81	19
Hematologic/Oncologic	15	8
Intoxication	92	0
Metabolic/Endocrine	29	6
Neurologic	223	19
Other	95	5
Postsurgical recovery	8	0
Respiratory	497	76
Sepsis	136	36
Shock	75	20

Table S2: Calibration analysis data

Calibration analysis (Hosmer-Lemeshow, Brier score)

Score	Hosmer-Lemeshow chi2	df	HL P value	Brier score	Calibration intercept (95% CI)	Calibration slope
pSOFA	42.096	8	<0.001	0.0942	0.000 (-0.300 to 0.300)	-
PRISM 4	231.81	8	<0.001	0.0886	0.933 (0.562 to 1.303)	0.87
PIM 3	631.371	8	<0.001	0.1069	0.231 (-0.115 to 0.577)	0.59

Calibration intercept = 0 and calibration slope = 1 indicate perfect calibration. Brier score: lower values indicate better overall probabilistic accuracy

pSOFA — decile-level observed vs. predicted

decile	N in decile	Deaths observed	Observed rate	Expected deaths	Expected rate
0	144	0	0	4.6964	0.0326
1	144	6	0.0417	6.1609	0.0428
2	144	0	0	7.5282	0.0523
3	144	14	0.0972	7.645	0.0531
4	144	0	0	10.3296	0.0717
5	144	7	0.0486	11.9642	0.0831
6	144	24	0.1667	14.2019	0.0986
7	144	20	0.1389	20.8776	0.145
8	144	35	0.2431	31.9198	0.2217
9	144	83	0.5764	73.6766	0.5116

PRISM 4 — decile-level observed vs. predicted

decile	N in decile	Deaths observed	Observed rate	Expected deaths	Expected rate
0	144	2	0.0139	0.3708	0.0026
1	144	6	0.0417	0.7505	0.0052
2	144	4	0.0278	1.0899	0.0076
3	144	5	0.0347	1.3064	0.0091
4	144	5	0.0347	1.755	0.0122
5	144	12	0.0833	2.1704	0.0151
6	144	7	0.0486	3.1393	0.0218
7	144	19	0.1319	5.0527	0.0351
8	144	34	0.2361	11.9067	0.0827
9	144	95	0.6597	63.1354	0.4384

PIM 3 — decile-level observed vs. predicted

decile	N in decile	Deaths observed	Observed rate	Expected deaths	Expected rate
0	144	2	0.0139	0.1879	0.0013
1	144	7	0.0486	0.3032	0.0021
2	144	4	0.0278	0.4873	0.0034
3	144	3	0.0208	1.0816	0.0075
4	144	9	0.0625	1.737	0.0121
5	144	14	0.0972	2.3693	0.0165
6	144	21	0.1458	3.2918	0.0229
7	144	39	0.2708	5.4664	0.038
8	144	30	0.2083	11.361	0.0789
9	144	60	0.4167	49.337	0.3426

Table S3. Decision curve analysis

Threshold probability	pSOFA net benefit	PRISM 4 net benefit	PIM 3 net benefit	Treat-all net benefit	Treat-none net benefit
1%	0.122474747	0.115074355	0.11523569	0.122474747	0
2%	0.113520408	0.100056689	0.097534014	0.113520408	0
3%	0.105927835	0.095260596	0.081207045	0.104381443	0
4%	0.097627315	0.088368056	0.067274306	0.095052083	0
5%	0.089875731	0.083881579	0.055116959	0.085526316	0
6%	0.086288416	0.080747636	0.050930851	0.075797872	0
7%	0.080719833	0.077897252	0.046863799	0.065860215	0
8%	0.083695652	0.075754831	0.044444444	0.055706522	0
9%	0.079739011	0.073534799	0.039537546	0.04532967	0
10%	0.072839506	0.072685185	0.038348765	0.034722222	0
11%	0.07025593	0.068812422	0.036204744	0.023876404	0
12%	0.067613636	0.067424242	0.035227273	0.012784091	0
13%	0.0649106	0.065229885	0.033053959	0.001436782	0
14%	0.057751938	0.060788114	0.031928295	-0.010174419	0
15%	0.055718954	0.058823529	0.02994281	-0.022058824	0
16%	0.053637566	0.058201058	0.029232804	-0.03422619	0
17%	0.051506024	0.05499498	0.02727577	-0.046686747	0
18%	0.043936314	0.053472222	0.024813686	-0.05945122	0
19%	0.042472565	0.052674897	0.023928326	-0.072530864	0
20%	0.040972222	0.050868056	0.022743056	-0.0859375	0
21%	0.039433896	0.049094585	0.020912447	-0.099683544	0
22%	0.037856125	0.047507123	0.019782764	-0.113782051	0
23%	0.039177489	0.04752886	0.020391414	-0.128246753	0
24%	0.038121345	0.047368421	0.019773392	-0.143092105	0

25%	0.037037037	0.046527778	0.019444444	-0.158333333	0
26%	0.035923423	0.044557057	0.019857357	-0.173986486	0
27%	0.0347793	0.042808219	0.018017504	-0.190068493	0
28%	0.033603395	0.041743827	0.016396605	-0.206597222	0
29%	0.032394366	0.040825509	0.016206964	-0.223591549	0
30%	0.026984127	0.039781746	0.01577381	-0.241071429	0
31%	0.026107085	0.038727858	0.015952093	-0.259057971	0
32%	0.025204248	0.036601307	0.015482026	-0.277573529	0
33%	0.024274461	0.03518864	0.014376036	-0.296641791	0
34%	0.023316498	0.035542929	0.015382997	-0.316287879	0
35%	0.02232906	0.034241453	0.014690171	-0.336538462	0
36%	0.021310764	0.033246528	0.014019097	-0.357421875	0
37%	0.018860229	0.031261023	0.013657407	-0.378968254	0
38%	0.018077957	0.027486559	0.0109319	-0.401209677	0
39%	0.017270036	0.026923953	0.009175774	-0.424180328	0
40%	0.016435185	0.02662037	0.009027778	-0.447916667	0
41%	0.015572034	0.025612053	0.008733522	-0.472457627	0
42%	0.014679119	0.025287356	0.007351533	-0.497844828	0
43%	0.013754873	0.024951267	0.006384016	-0.524122807	0
44%	0.012797619	0.02514881	0.006646825	-0.551339286	0
45%	0.008522727	0.023421717	0.006944444	-0.579545455	0
46%	0.00784465	0.023070988	0.006584362	-0.608796296	0
47%	0.007140985	0.021239518	0.006878931	-0.639150943	0
48%	0.006410256	0.020138889	0.005982906	-0.670673077	0
49%	0.005650871	0.019798475	0.00577342	-0.703431373	0
50%	0.004861111	0.01875	0.00625	-0.7375	0
Net benefit = (TP/n) - (FP/n)*(pt/(1-pt)) at each threshold probability pt, standard Vickers & Elkin (2006) decision curve analysis formula. All three scores showed positive net benefit relative to the treat-none strategy across the evaluated 1%-50% threshold range. Relative to the treat-all strategy, net benefit became greater from approximately 3% for pSOFA, 6% for PRISM 4, and 10% for PIM 3					

Table S4: Sensitivity analyses

Analysis	N	Deaths	Mortality %	pSOFA AUC	PRISM4 AUC	PIM3 AUC
Primary (n=1440, all encounters)	1440	189	13.12	0.8067	0.8467	0.7813
Sensitivity: first encounter only	1133	143	12.62	0.826	0.8639	0.7912
Sensitivity: excl. age protocol violations	1438	189	13.14	0.8065	0.8466	0.7814
Sensitivity: excl. mvdays>365 outliers	1422	188	13.22	0.8069	0.8467	0.7824
All three AUCs are stable within ±0.02 across every sensitivity check						
